# STUDIES OF MOLECULAR CHANGES IN INTERVERTEBRAL DISC DEGENERATION IN ANIMAL MODEL

**DOI:** 10.1590/1413-785220162401152960

**Published:** 2016

**Authors:** Marcelo Ferraz de Campos, Cintia Pereira de Oliveira, Charles Benjamin Neff, Olga Maria de Toledo Correa, Maria Aparecida Silva Pinhal, Luciano Miller Reis Rodrigues

**Affiliations:** 1. Faculdade de Medicina do ABC, Orthopedics Discipline, Santo André, SP, Brazil.; 2. Faculdade de Medicina do ABC, Biochemistry Department, Santo André, SP, Brazil.; 3. Faculdade de Medicina do ABC, Cell Biology Department, Santo André, SP, Brazil.; 4. Universidade Federal de São Paulo, Biochemistry Department, São Paulo, SP, Brazil.

**Keywords:** Intervertebral disc degeneration, Collagen, Metalloproteases, Neovascularization, pathologic, Apoptosis

## Abstract

**Objective:**

To evaluate the structural and molecular changes in the extracellular matrix (ECM) during the process of intervertebral disc degeneration, using animal model.

**Methods:**

Wistar rats underwent intervertebral disc degeneration through 20-gauge needle puncture, and 360° rotation applied for 30 sec, representing the degenerated group, while control group was not submitted to this procedure. Histological parameters and expression of extracellular matrix molecules were evaluated in the 15^th^ and 28^th^ days after degenerative induction.

**Results:**

Fifteen days after the induction of intervertebral disc degeneration, significant changes were observed, such as reduction in the expression metalloprotease-9 (MMP9) and interleukins (IL-6 and IL-10). There was a significant increase in the expression of vascular endothelial growth factor (VEGF) and caspase-3. However, different alterations in the ECM were observed at 28 days, the level of collagen I, metalloprotease-2 (MMP2) and caspase-3 were enhanced. Furthermore, expression of heparanase isoforms (HPSE1 and HPSE2) mRNA were increased in the degenerative intervertebral disc.

**Conclusion:**

The different profiles of ECM molecules observed during the intervertebral disc degeneration suggest that molecular processes such as ECM remodeling, neovascularization, apoptosis and inflammation occur. ***Experimental Study.***

## INTRODUCTION

The intervertebral disc degeneration (IDD) may play an important role in the chronic evolution of the back pain, but still needs greater elucidation of clinical and molecular mechanisms. Intervertebral disc degeneration results in the structural changes to the extracellular matrix. ^1^ ^-^ ^3^

Metalloproteases (MMP) and heparanase (HPSE) are enzymes involved in degradation of the extracellular matrix molecules and play an important role in the process of intervertebral disc degeneration. ^4^ ^-^ ^6^It was describe that MMP2 mediates local degradation and of collagen in the intervertebral disc. ^7^Heparanase-1 is an endo=beta-glucuronidase that degrades heparin sulfate chain from proteoglycans generating oligosaccharides that enhance the response of growth factors, angiogenic factors and cytokines. However, heparanase-2 (HPSE2) has no enzymatic activity, but present a 30% homology with HPSE1 and its biological role still unknown. ^5^ ^,^ ^6^

The cyokines IL-6 and IL-10 respectively, an anti-inflammatory and pro-inflammatory cytokine are also involved in disc degeneration processes. ^8^ ^-^ ^10^

During disc degeneration neurovascular structures may be induced by the inflammatory and angiogenic factors such as the VEGF. ^11^

Moreover, apoptotic events occurring in the nucleus pulposus cells seem to be mediated by caspases. ^12^ ^,^ ^13^

In the present study, we evaluated the expression and distribution of structural and molecular constituents of the ECM such as collagen, metalloproteases, glycosidases (heparanase-1 and heparanase-2), cytokines, VEGF and caspase-3 in the intervertebral disc during degenerative process to elucidate alterations that may be involved with the degenerative process and to better understand the pathophysiology of the disease, contributing towards future improvements in treatment approaches as an attempt to intervene or prevent disease progression.

## MATERIALS AND METHODS

This study was approved by the Ethics in Animal Experimentation Committee of the *Faculdade de Medicina do ABC*, process # 003/2011. 

For the animal model of degeneration of the intervertebral disc, 12 male Wistar rats were use ( *Rattus norvergicus albinus*), from the Animal House of the Faculdade de Medicina do ABC, Santo André, SP, Brazil, at 12 weeks of age (complete skeletal maturity), weighing between 300 and 350g. The rats were separated into three groups. Two animals were not submitted to degeneration induction and were euthanized at 28 days, representing the control group. The second group of five animals was euthanized 15 days after induction of the intervertebral disc degeneration process, and the third group of five animals was euthanized 28 days after the degeneration induction. The animals remained under the care of the animal house of the Medical School after the induction of degeneration until the time of euthanasia. 

Antisepsis was performed on the animal tail with a solution of alcohol iodate; then the animals were anesthetized with an association of ketamine (88 mg/kg) and xylazine 2% (12 mg/kg) by intraperitoneal route. The deep anesthetic plane was confirmed by the absence of corneal reflex and by the absence of reaction to deep pain provoked by the compression of the interdigits of the paws. The levels between the sixth and seventh, seventh and eighth, and eighth and ninth coccygeal vertebrae were identified by radioscopy. Induction of degeneration was performed by percutaneous puncture with a 20G needle. The needle was introduced until it reached the nucleus pulposus, turned 360°, and maintained in the same position for 30 seconds, as described in the literature. ^14^ ^,^ ^15^

The samples were collected after 15 and 28 days after the puncture. For the euthanasia, the animals were deep anesthetized and 5 mL of arterial blood were collected from the abdominal aorta, via trans-abdominal access, causing the euthanasia by hypovolemic shock. After the euthanasia, the samples were removed and stored in RNA Holder or stored in formaldehyde (10%), respectively, gene expression and protein expression studies. 

Slices with 3 µm in thickness, embedded in paraffin and fixed in formalin, were deparaffinized and rehydrated. The recovery of the antigen was performed by warming of the slides at 100ºC, for 30 minutes in 10 mmol/L citrate buffer, pH 6.0. Activity of the endogenous peroxidase was blocked with an aqueous solution of 3% hydrogen peroxide for 35 minutes. The samples were incubated overnight at 4ºC with the primary antibodies: anti-MMP-2 (H-76), anti-MMP-9 (H-129), anti-interleukin-6 130326, and anti-interleukin-10 (H-160) (Santa Cruz Biotechnology, USA), anti-caspase-3 (3015-100) (BioVision, USA) anti-collagen I (C 2456) (Sigma, USA), and anti-VEGF-A (18077) (Biorbyt, England). Finally, the slides were incubated with a complex of streptavidin marked with peroxidase followed manufacture instructons (LSAB^(r)^, Dako Cytomation, Glostrup, Denmark). The sections were developed, using 3,3'-diaminobenzidine (DAB) counterstained with hematoxylin. The presence of a brown color was considered evidence of positive expression of the respective molecules. Picrosirius Red stain was done the images were analyzed with normal light and polarized light for the study of collagen fibers. 

The slides were analyzed with the help of a TS100 Nikon Eclipse^(r)^ light microscope to identify the areas that best represented the immune marking of the molecules analyzed (hot spots). In each case, quantification of the immune labelling was quantified by a digital analysis as described below. The photomicrographs with 640x480 pixels were obtained from consecutive non-coincident fields with 400X magnification using a 4300 Nikon Coolpix^(r) ^digital camera adjusted for the same parameters. The images obtained were analyzed by the system of processing and analysis of images of ImageLab^(r)^ (Softium Informática^(r)^, São Paulo, Brazil), adjusted for a micrometric scale (µm), as decribed by Matos et al. ^16^

The digital quantification was expressed by the index of expression (IE). Index of expression was obtained by the multiplication of the percentage of stained cells (PI) by the intensity of digital immune staining (ItE) for each sample, as described in the following equation and 



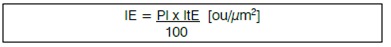



### mRNA expression by quantitative RT-PCR

Total RNA was obtained using the Trizol^(r)^ reagent (Ambion by Life Technologies(tm), CA, USA), following the instructions of the manufacturer. RNA quantification was determined on the NanoVue Plus device (GE Healthcare, Germany). The reverse transcription was performed using the reverse transcriptase enzyme ImPromII(tm) (Promega Co., WI, USA), as per the manufacturer's instructions to obtain the complementary DNA (cDNA). The cDNA obtained in the reverse transcriptase reaction was used for the amplification of the isoforms of heparanase (HPSE1, HPSE2). The expression of mRNA was represented as relative value using constitutive endogenous genes, glyceraldehyde-3-phosphate-dehydrogenase (GAPDH), thus determining the values of (-ΔCt). The expression of target-genes was analyzed using the primers for the isoforms of heparanase (HPSE1, HPSE2) and GAPDH sequences, described in the Table 1. The assays were performed in triplicate. All the primers were produced by Applied Biosystems, CA, USA. Amplification was performed using the reagent Maxima^(r)^ SYBR Green qPCR Master Mix (2X) (Applied Biosystems, CA, USA), following the manufacturer protocol. The reaction was submitted to a thermocycler for real-time amplification (7500 Real Time PCR Cycler^(r))^ (Applied Biosystems, CA, USA), with cycling of 95ºC per 10 minutes, followed by 40 cycles (95ºC, 15 seconds; 60ºC, 60 seconds). 

**Table 1:** Oligonucleotide sequences used as primers.

mRNA	Forward	Reverse
GAPDH	5'TCTAGAGACAGCCGCATCTTCTTG3'	5'GTCCGATACGGCCAAATCCGTTCA3'
HPSE1	5'AGAAGTCGTGATGAGGCAGGTGTT3'	5'TTGGGTGATAGACGTTCGTGCAGT3'
HPSE2	5'TTCTAGTGCCCTGAGCCTGTTGAA3'	5'TCCCAACTGACTGCCATTTACTGC3'

### Statistic Analysis

The quantitative statistical analysis was performed using the GrandPad Prism 5.0 program (La Jolla, CA, USA); to verify the occurrence of significant differences between the quantitative variables, Kruskal-Wallis's non-parametric test with Dunn's supplementary test were used in comparisons of subgroups. For the parametric analysis, the Chi-squared test was used to evaluate the qualitative variables using the SPSS program version 17.0 (SPSS, Chicago, IL, USA). In all analyses, a 5% significance level was adopted (p£0.05).

## RESULTS

Histologic and pathologic parameters evaluation are described in the Table 2. Concerning cellular alterations, it can be observed no apoptotic events in the control group, while all samples of the degenerative intervertebral discs presented apoptosis. Moreover, there was a significant difference between degenerative samples, since apoptotic events were more intense in the samples obtained from 28 days compared to the samples in the 15 days samples (p=0.002). Furthermore, no regenerative process was verified in the control group. However, mild and moderate regeneration was present in the samples obtained from animals submitted to disc degeneration process (p=0.01).

**Table 2:** Histologic features during intervertebral disc degeneration.

Histological features		Control (n=2)	Degeneration 15 days (n=5)	Degeneration 28 days (n=5)	p
Cellulat alterations	Apoptosis	*0.002
(0)	2 (100.0)	0 (0.0)	0 (0.0)
(+)	0 (0.0)	3 (60.0)	0 (0.0)
(++)	0 (0.0)	2 (40.0)	5 (100.0)
(+++)	0 (0.0)	0 (0.0)	0 (0.0)
Regeneration	*0.010
(0)		2 (100.0)	0 (0.0)	0 (0.0)
(+)		0 (0.0)	5 (100.0)	4 (80.0)
(++)		0 (0.0)	0 (0.0)	1 (20.0)
(+++)		0 (0.0)	0 (0.0)	0 (0.0)
Extracellular matrix alterations	Fracture/fissure	0.186
	(0)		2 (100.0)	3 (60.0)	5 (100.0)
	(+)		0 (0.0)	2 (40.0)	0 (0.0)
	(++)		0 (0.0)	0 (0.0)	0 (0.0)
	(+++)		0 (0.0)	0 (0.0)	0 (0.0)
	Calcification	*0.022
	(0)		2 (100.0)	1 (20.0)	0 (0.0)
	(+)		0 (0.0)	0 (0.0)	2 (40.0)
	(++)		0 (0.0)	1 (20.0)	3 (60.0)
	(+++)		0 (0.0)	3 (60.0)	0 (0.0)
	Mixoid degeneration	0.065
	(0)		1 (50.0)	0 (0.0)	0 (0.0)
	(+)		1 (50.0)	5 (100.0)	5 (100.0)
	(++)		0 (0.0)	0 (0.0)	0 (0.0)
	(+++)		0 (0.0)	0 (0.0)	0 (0.0)
	Eosinophilic degeneration	0.125
	(0)		2 (100.0)	1 (20.0)	0 (0.0)
	(+)		0 (0.0)	2 (40.0)	2 (40.0)
	(++)		0 (0.0)	1 (20.0)	3 (60.0)
	(+++)		0 (0.0)	1 (20.0)	0 (0.0)
	Inflammatory infiltrate	*0.025
	(0)		2 (100.0)	1 (20.0)	1 (20.0)
	(+)		0 (0.0)	0 (0.0)	4 (80.0)
	(++)		0 (0.0)	3 (60.0)	0 (0.0)
	(+++)		0 (0.0)	1 (20.0)	0 (0.0)
	Neovascularization	0.380
	(0)		2 (100.0)	1 (20.0)	1 (20.0)
	(+)		0 (0.0)	1 (20.0)	2 (40.0)
	(++)		0 (0.0)	2 (40.0)	2 (40.0)
(+++)	0 (0.0)	1 (20.0)	0 (0.0)

Degree of alterations: 0, absent; (+), Low; (++), Mild; (+++), Intense; n, number of rats.

Percentage (%), * Statistic Significance (Qui-Square test).

Significant differences were also observed in the extracellular matrix of the intervertebral disc. Intense calcification and inflammatory infiltrate were present at 15 days after degenerative process, whilst such events represented moderate level in the samples obtained at 28 days, respectively, p = 0.022 and p = 0.025. Additionally, none of these events were detected in the control group. There were no significant differences in the analysis of fractures and fissures, myxoid and eosinophilic degeneration between the groups. ( Table 2)

In the process of neovascularization of the disc, we observed the noteworthy presence of new vessels during the process of degeneration of the intervertebral disc and absent in the control group, despite these results are not statistically significant as shown in the Table 2.

**Figure 1:**
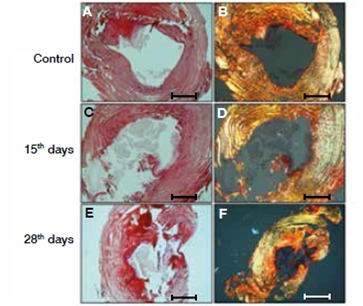
Analysis of collagen distribution. Picrosirius red staining was used to evaluate collagen fibers of the annulus fibrosus (AF), under normal and polarized light, during intervertebral disc degenerative process. A, C and E normal light evaluation; B,D and F polarized light; 28 days, AF obtained 28 days after degenerative induction; 25 days, AF obtained 25days after degenerative induction. Fibers association (red); new fibrils formation (green).

In the control group we observed that the collagen fibers of the AF had a pattern of organization, with only longitudinal strands oriented along a single direction. The fibers were thickened and densely packed. ( Figures 1A and B) At 15 days of degeneration of the intervertebral disc, we observed structural changes in the fibers, with the presence of longitudinal and transversal strands with no pattern of organization, and fibers were not as thick relative to the controls ( Figures 1C and D). At 28 days of degeneration, we noted a high degree of strand disorganization, presence of fibrils represented with green coloring under polarized light, and an association of new collagen fibers represented in red. ( Figures 1E and F)

**Figure 2:**
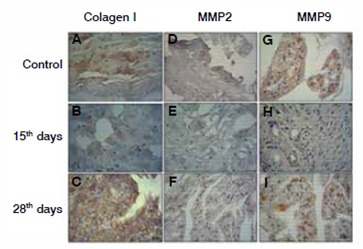
Collagen I, metalloprotease-2 (MMP2) and metalloprotease-9 (MMP9) Expression. Immunohistochemistry reaction in the intervertebral disc after degeneration (15 and 28 days), control (samples obtained from rats non submitted to the degenerative process). A, B and C, Collagen I; D, E and F, MMP2; G, H and I, MMP9).

Analysis of the expression of collagen I revealed a significant increase at 28 days relative to the degenerated group. ( Figures 2and ^3^A)

**Figure 3:**
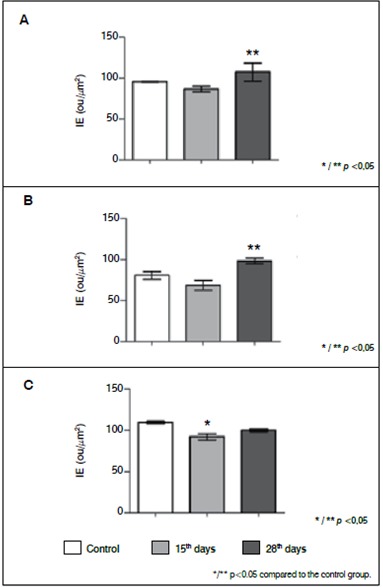
Digital quantification of collagen I, metalloprotease-2 (MMP2) and metalloprotease-9 (MMP9). (A) collagen I; (B) MMP2; (C) MMP0. The values represent the Index of Expression (IE) obtained as described in methods. The values represent media and standard deviation. The statistic analysis were performed using Kruskal-Wallis Test with Dunn's Multiple Comparison Test.

The protein expression of metalloprotease MMP9 is decreased at 15 days of disc degeneration. On the other hand, a significant increase of MMP2 was noted after 28 days of intervertebral disc degeneration as demonstrated in Figure 2, Figures 3B and C.

**Figure 4:**
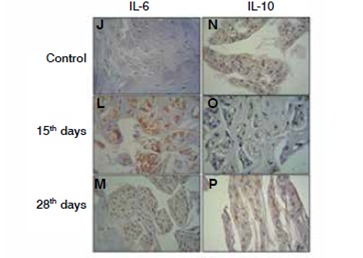
IL-6 and IL-10 Immunoexpression Immunohistochemistry reaction in the intervertebral disc after degeneration (15 and 28 days), control (samples obtained from rats non submitted to the degenerative process). J, L and M, Interleukin-6 (IL-6); N, O and P, interleukin-10 (IL-10).

Figures 4and ^5^show, respectively, the immunohistochemistry reaction and digital quantification for evaluation of the inflammatory process of the intervertebral disc by the analysis of interleukins profile. An increase in expression of IL-6 was noted during the degenerative process along with a decrease of IL-10 restricted to 15 days of degenerative process. 

**Figure 5:**
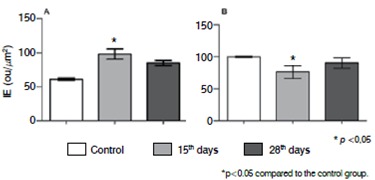
Digital quantification of IL-6 and IL-10. The values represent the Index of Expression (IE) obtained as described in Methods. The values represent media and standard deviation. (A) IL-6; (B) IL-10. The statistic analysis were performed using Kruskal-Wallis Test with Dunn's Multiple Comparison Test.

**Figure 6:**
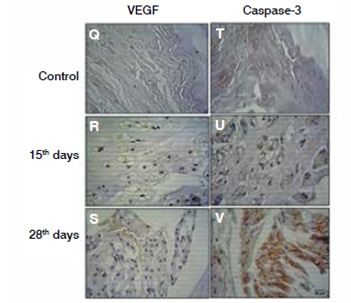
VEGF and caspase-3 immunoexpression. immunohistochemistry reaction in the intervertebral disc after degeneration (15 and 28 days), control (samples obtained from rats non submitted to the degenerative process). Q, R and S, VEGF; T, U and V caspase-3.

**Figure 7:**
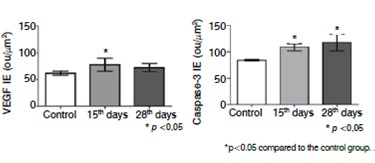
Digital quantification of VEGF e Caspase-3. The values represent the Index of Expression (IE) obtained as described in methods. The values represent media and standard deviation. The values represent media and standard deviation. The statistic analysis were performed using Kruskal-Wallis Test with Dunn's Multiple Comparison Test.

In the analysis of the expression of VEGF, the vascular endothelial growth factor that may be involved in the neovascularization, we noted a significant increase 15 days after the disc degeneration relative to the controls. ( Figure 6) Digital quantification confirmed the results obtained by immunohistochemistry. ( Figure 7) 

The values obtained by the index of expression (IE) of caspase-3, a protease directly involved with mechanisms of cellular apoptosis, increased significantly during the degenerative process of the intervertebral disc at 28 days compared to the control group. ( Figures 6and ^7^) 

**Figure 8:**
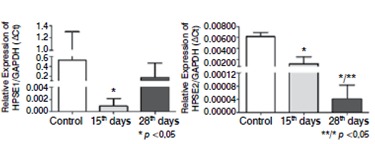
Heparanase-1 (HPSE1) and heparanase-2 (HPSE2) expression by quantitative RT-PCR. Quantitative RT-PCR analysis were performed as described in Methods, using cDNA samples obtained from intervertebral disc after degeneration induction (15 and 28 days), control (samples obtained from rats non submitted to the degenerative process).

The expression of the messenger RNA (mRNA) of the isoforms of heparanase was carried out by quantitative RT-PCR analysis, which demonstrated that the degenerated intervertebral discs presented a significant reduction in the expression of HPSE1 and HPSE2 after the induction of the degenerative process when compared to the control group, as is shown in Figure 8.

## DISCUSSION

In the histopathologic evaluation of the degenerative intervertebral samples of rats we noted evident cellular alterations such as apoptosis that increased gradually during the development of the degenerative process (14 and 28 days of induction of degeneration), mild regeneration, intense calcification and moderate presence of inflammatory infiltrate. The study done by Haschtmann and coworkers demonstrated that in the degenerative intervertebral disc of the rat model, the proportion of cells in apoptosis also increased significantly, corroborating our results. ^17^

The degeneration of the intervertebral disc altered significantly the structural organization of the collagen, since degenerative samples presented disordered fibers that are not as thick as control samples representing new collagen fibers, beyond to increase type I collagen expression. 

It is known that MMP2 has more expression in degenerative processes and that it participates directly in remodeling of the collagen by regulating the activity of the gelatinases. ^7^These data corroborates with our results, since MMP2 protein expression increased at 28 days in the degenerative discs together with the disorganization of collagen. 

It is acknowledged that metalloproteases are involved with extracellular matrix remodeling. ^4^ ^,^ ^18^The results demonstrated higher level MMP2 and MMP9 in the degenerative intervertebral disc compared to the samples obtained by non-affected tissue, suggesting their role in extracellular matrix remodeling. 

 Inflammatory cytokines have a fundamental role in the phases of degeneration of the intervertebral disc. The start of the disease is characterized by an initial lesion where the cells regulate the expression of inflammatory cytokines such as TNF, IL - 1β, and IL-6, as well as degradation molecules of the extracellular matrix, resulting in tears, fissures causing mechanical instability to the disc. In the second phase of the disease, the cytokines are released activating the infiltration of leukocytes into the tissue and the inflammatory response is accompanied by neovascularization and the appearance of nervous fibers. The final phase is characterized by sensitization of the nervous endings mediated by the inflammatory process and neurotrophins resulting in pain. ^8^ ^,^ ^9^

 In our study, we obtained a significant increase in the protein expression of IL-6 an anti-inflammatory cytokine, while there was a significant decrease of IL-10 that is a pro-inflammatory cytokine at the same time (15 days) of disc degeneration. The histologic evaluation of the tissues showed a moderate level of inflammatory process, which may suggest a balance between the action of such interleukins. Moreover, the abnormal production of pro-inflammatory molecules in the degenerative process of the disc can trigger a series of pathogenic responses in the cells of the intervertebral disc promoting autophagy, senescence, and cellular apoptosis. ^19^Therefore, the increased level of IL-10 can be modulating apoptosis events in the degenerative discs. 

VEGF expression are enhanced at 15 days after the intervertebral disc degeneration and neovascularization was also present in the degenerative samples and absent in the control samples (non affected by disc degeneration). Furthermore, taken together these results are consistent with those of literature. ^8^VEGF is involved in processes of abnormal neovascularization, growth of vessels in symptomatic discs. There data corroborate literature, in which one study with human cells showed that a lesion in AF has the potential to initiate an inflammatory process and the neovascularization of the tissue involving a VEGF and inflammatory cytokines IL-6, IL-8, and TGF-β. ^20^

Caspase-3 represents a marker of apoptosis induction and its expression increases gradually during the degenerative process of intervertebral disc ^12^which is consistent with the high levels of caspase-3 during the entire degenerative process in rats, obtained in our results. 

High level of HPSE1 obtained in the samples submitted to intervertebral disc degeneration indicates that this enzyme is possibly involved with the processes of tissue remodeling during degenerative process. Our data confirm that the isoform HPSE2 are also possibly involved in the development of intervertebral disc degeneration, due to increased expression compared to the non-degenerative discs.

## CONCLUSION

The alteration of metalloproteases, collagen, glycosidases, VEGF, caspase-3, and interleukins observed in the present study, suggest an intense remodeling process of the extracellular matrix of the degenerative intervertebral discs.

Better understanding of the molecular mechanisms involved in the intervertebral disc degeneration is important to elucidate the pathophysiology of the degenerative disc disease that affects the population worldwide and directly impacts the quality of life of the individuals.
